# Perivascular Adipose Tissue and Vascular Smooth Muscle Tone: Friends or Foes?

**DOI:** 10.3390/cells12081196

**Published:** 2023-04-20

**Authors:** Amer Ahmed, Aasia Bibi, Massimo Valoti, Fabio Fusi

**Affiliations:** 1 Department of Life Sciences, University of Siena, Via Aldo Moro 2, 53100 Siena, Italy; aa.biotechiub@gmail.com (A.A.); massimo.valoti@unisi.it (M.V.); 2 Nanotechnology Institute, CNR-NANOTEC, Via Monteroni, 73100 Lecce, Italy; aasiabibi250@gmail.com; 3 Department of Biotechnology, Chemistry and Pharmacy, University of Siena, Via Aldo Moro 2, 53100 Siena, Italy

**Keywords:** PVAT, vascular tone, anti-contractile, pro-contractile, endothelial dysfunction

## Abstract

Perivascular adipose tissue (PVAT) is a specialized type of adipose tissue that surrounds most mammalian blood vessels. PVAT is a metabolically active, endocrine organ capable of regulating blood vessel tone, endothelium function, vascular smooth muscle cell growth and proliferation, and contributing critically to cardiovascular disease onset and progression. In the context of vascular tone regulation, under physiological conditions, PVAT exerts a potent anticontractile effect by releasing a plethora of vasoactive substances, including NO, H_2_S, H_2_O_2_, prostacyclin, palmitic acid methyl ester, angiotensin 1-7, adiponectin, leptin, and omentin. However, under certain pathophysiological conditions, PVAT exerts pro-contractile effects by decreasing the production of anticontractile and increasing that of pro-contractile factors, including superoxide anion, angiotensin II, catecholamines, prostaglandins, chemerin, resistin, and visfatin. The present review discusses the regulatory effect of PVAT on vascular tone and the factors involved. In this scenario, dissecting the precise role of PVAT is a prerequisite to the development of PVAT-targeted therapies.

## 1. Introduction

Blood vessels are comprised of three layers, namely *tunica intima*, *tunica media,* and *tunica adventitia*, organized from the innermost to the outermost side and consisting mainly of endothelial cells, vascular smooth muscle cells (VSMCs), and fibroblasts, respectively. The majority of blood vessels, except cerebral and pulmonary arteries, are surrounded by a fourth layer of adipose tissue called perivascular adipose tissue (PVAT) or *tunica adiposa* [1,2].

Understanding the regulation of vascular tone is crucial to cardiovascular physiopathology. Normal vascular tone is fine-tuned by the autonomic nervous system, hormones, and by autocrine and paracrine factors produced by adjacent blood vessel layers, i.e., endothelium and PVAT [3,4,5]. PVAT, a specialized type of adipose tissue surrounding blood vessels, was traditionally thought to act merely as a mechanical support adhering blood vessels to other tissues/organs; therefore, it is usually removed during in vitro vascular studies [6]. In the early 1990s, a pioneered study by Soltis and Cassis [7] showed that the presence of PVAT surrounding thoracic rat aorta rings attenuated noradrenaline-induced contraction; this effect was ascribed to catecholamine re-uptake into adrenergic nerves [7]. The anti-contractile effect of PVAT was later observed with other vasoconstrictors (i.e., serotonin, angiotensin II, and phenylephrine) and transferring the solution from the organ bath of a PVAT-intact preparation (donor) to that of a PVAT-deprived preparation (recipient) caused a noticeable relaxation of the vessel tone; eventually, the term adipocyte-derived relaxing factor (ADRF) was coined [8,9]. Several reports attempted to identify the nature of ADRF and delineate the mechanisms underpinning its anti-contractile effect [10]. As a result, discrete factors such as gases (NO and H_2_S), small molecules [(H_2_O_2_, prostacyclin, and palmitic acid methyl ester (PAME)], and proteins or peptides (leptin, apelin, angiotensin 1-7, adiponectin, and omentin) were associated with PVAT anti-contractile effect [11,12].

Meanwhile, several studies showed that PVAT, under certain pathophysiological conditions such as obesity, hypertension or diabetes, loses its anti-contractile effect and even potentiates blood vessel contraction, thus exerting deleterious effects on the vasculature [13,14,15,16]. Similarly to the anti-contractile effect, PVAT pro-contractile effect seems to be mediated by several factors, including superoxide anion, catecholamines, prostaglandins, angiotensin II, chemerin, leptin, resistin, and visfatin [14]. PVAT is now well recognized for its contribution to the regulation of vascular tone via outside-in signalling [3,5]. Moreover, PVAT is involved in all aspects of vascular pathophysiology because it modulates inflammation-associated vascular pathologies such as vascular remodelling, atherosclerosis, and obesity by secreting several adipokines, pro- and anti-inflammatory cytokines, and chemokines (for a review, see [17,18,19]).

As a result of this double-edged sword nature, PVAT is considered a unique and, at the same time, fundamental therapeutic target for the treatment of cardiovascular diseases. This review aims at providing a comprehensive overview of the factors released from PVAT and their role in the regulation of vascular tone in an attempt to highlight their potential as drug targets/candidates. The role of PVAT in the regulation of VSMC growth (reviewed in [18,20]) and in the pathogenesis of cardiovascular diseases (reviewed in [21,22,23]) is beyond the scope of the present review and is not discussed.

## 2. Characteristics of Perivascular Adipose Tissues

**Figure 1:**
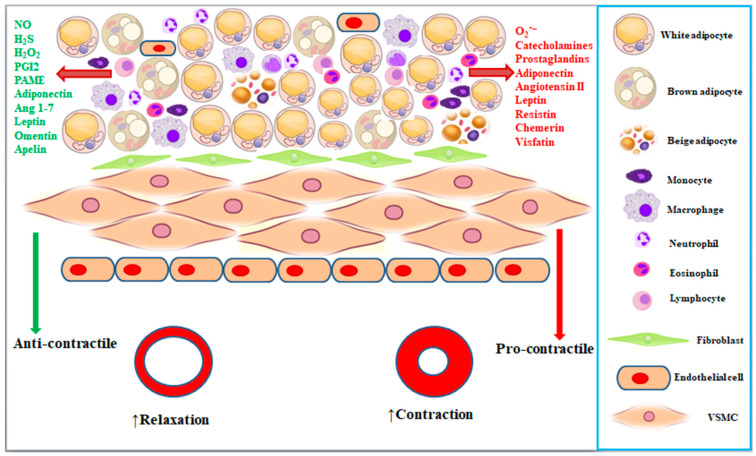
Blood vessels are made up of three cellular layers, namely *intima* (endothelial cells), *media* (VSMCs) and *adventitia* (fibroblast), and are surrounded by a fourth layer of adipose tissue called PVAT. PVAT is characterized by cellular heterogeneity harbouring adipocytes, endothelial and immune cells. PVAT regulates vascular tone via the secretion of vasoactive substances and adipokines. These factors, either anti-contractile or pro-contractile, decrease or increase the contractility of VSMCs, respectively. Ang 1-7, Angiotensin 1-7; PAME, palmitic acid methyl ester; PGI2, prostacyclin I_2_; VMSC, vascular smooth muscle cell.

PVAT displays several characteristics that distinguish it from other blood vessel layers (*intima*, *media*, and *adventitia*) or adipose tissues. First, PVAT is not separated from the underlying blood vessel by a physical layer or an elastic lamina being rather in direct contact with the *tunica adventitia* [24]. This allows bioactive substances and adipokines released from PVAT to directly affect blood vessels in a paracrine mode, though an endocrine mode may also occur via the *vasa vasorum* present within PVAT itself [25]. Similarly, the signalling molecules originating in the vasculature diffuse in an inside–outside manner toward PVAT, where they modulate its secretory function [26]. In fact, the direct contact of PVAT with vasculature seems essential for its regulatory role as PVAT detached from blood vessels loses its anti- and/or pro-contractile effect [11]. Second, unlike *tunica intima* and *tunica media,* which are formed solely of endothelial cells and VSMCs, respectively, but likewise other adipose tissues, PVAT hosts pre- and mature adipocytes, endothelial cells, mesenchymal stem cells, and immune cells, including monocytes, macrophages, mast cells, eosinophils, and lymphocytes (T and B cells), though adipocytes are the predominant cells (Figure 1) [27,28]. The cellular heterogeneity may be altered under certain pathological conditions, such as inflammation, characterized by increased infiltration of macrophages [29,30,31]. Third, PVAT displays phenotypic heterogeneity depending on the vascular bed, species, age, and health/disease status. For instance, PVAT surrounding the rodent thoracic aorta exhibits a brown adipose tissue phenotype, and PVAT surrounding the abdominal aorta and coronary arteries is a mixture of white and brown adipose tissues, whereas that surrounding the mesenteric, femoral, and carotid arteries resembles white adipose tissue [32,33]. This makes white PVAT of crucial importance because small vessels play a major role in the regulation of blood pressure compared to large vessels. Nevertheless, PVAT may either harbour beige adipocytes or undergoes beiging under certain pathophysiological stimuli [19]. Conversely, in adult humans, brown-like PVAT is nearly absent, and PVAT exhibits a white adipose tissue phenotype [24]. Fourth, PVAT adipocytes may originate from different precursors depending on their location [32]. For instance, periaortic arch adipocytes differentiate from ectoderm-derived neural crest cells [34]. Aortic (both thoracic and abdominal) and mesenteric PVAT was thought to originate from VSMC precursors since the deletion of peroxisome proliferator-activated receptor gamma (PPAR-γ, the master regulator of adipogenic differentiation) in VSMCs results in a complete loss of PVAT in both thoracic and abdominal aorta, as well as in mesenteric arteries [35]. Ye et al. [36] showed that anterior thoracic aortic PVAT is derived from SM22α^+^ progenitor cells, whereas lateral (right and left) aortic PVAT is from SM22α^+^ and Myf5^+^ progenitor cells. However, thoracic aorta PVAT adipocytes were recently demonstrated to originate from fibroblastic progenitor cells rather than VSMCs, thus challenging previous studies [37].

## 3. Perivascular Adipose Tissue Regulates Vascular Tone

Since the seminal study by Soltis and Cassis [7] demonstrating that thoracic aorta PVAT attenuates the response to noradrenaline, several studies have shown that PVAT decreases vascular contraction induced by various vasoconstricting agents in different vascular beds and species [8,38,39,40,41,42,43]. This function was ascribed to the release of ADRFs [8]. In fact, transferring the supernatant from PVAT-intact vessels (donors) to preparations deprived of PVAT (acceptors) caused a significant relaxation [44]. The release of ADRF was dependent on extracellular Ca^2+^ as well as protein tyrosine kinase and protein kinase A [45]. So far, it has been demonstrated that PVAT releases several anti-contractile and pro-contractile factors to regulate vascular tone (Figure 1). Anti-contractile factors [also known as PVAT-derived relaxing factors (PDRFs)] include gasotransmitters (NO and H_2_S), small molecules [H_2_O_2_, PGI2, and PAME], and adipocytokine (leptin, angiotensin 1-7, apelin, adiponectin, and omentin). These factors mediate the PVAT anti-contractile effect via endothelium-dependent and -independent mechanisms [9]. At the VSMC level, cGMP-dependent protein kinases (PKG) [46], as well as large-conductance Ca^2+^-activated [47], ATP-sensitive [8], and XE991-sensitive voltage-gated K^+^ channels [48] play a critical role in the regulatory activity of PVAT. In contrast, pro-contractile factors [(also known as PVAT-derived contracting factors (PDCFs)] include superoxide anion, catecholamines, prostaglandins, chemerin, angiotensin II, resistin, and visfatin (Figure 1). These factors, released from PVAT under certain physiological or pathological conditions such as obesity, hypertension, and diabetes, may directly or indirectly contribute to the pro-contractile effect of PVAT [14] by activating VSMCs Rho-kinase [49], inhibiting K^+^ channels [49,50], activating Ca_V_ channels [49,51], and increasing ROS generation. Noticeably, PVAT pro-contractile and anti-contractile effects, as well as the underpinning mechanisms, depend on the stimulus applied [38,44], vascular bed [52], gender [53], age [54], and health conditions of the model used [55]. The following sections discuss PVAT-derived anti-contractile and pro-contractile factors and briefly highlight their mechanism of action.

## 4. PVAT-Derived Anti-Contractile Factors

### 4.1. Nitric Oxide

NO, a gasotransmitter and potent vasodilator produced mainly by endothelial cells, regulates vascular tone and blood homeostasis [56]. Though Löhn et al. [8] showed that the anti-contractile effect of PVAT is not dependent on NO synthesis, numerous studies subsequently demonstrated that NO is produced within PVAT and contributes to its anti-contractile effect in different vascular beds, particularly in small arteries [13,38,57,58,59]. Furthermore, endothelial NO synthase (eNOS) is expressed in PVAT adipocytes and endothelial cells of *vasa vasorum* within PVAT [60,61,62]. Interestingly, neuronal NOS (nNOS) is also expressed in PVAT and contributes to its anti-contractile effect [38,63]. NOS expression and its contribution to PVAT anti-contractile effect vary depending on the vascular bed considered [64]. In Wistar rats, for example, eNOS expression is significantly lower in the abdominal compared to thoracic PVAT [52]. In mouse second-order mesenteric and *gracilis* arteries, PVAT reduces the response of electrical field stimulation and noradrenaline in nNOS- and eNOS-dependent manner, respectively. This effect is blunted in obese mice and can be restored by non-specific activation of NOS [38]. Noradrenaline stimulation of adipocyte β_3_-adrenoceptors activates the Gα_s_ signalling pathway leading to increased cAMP levels and the release of adipocyte-derived NO [38,44]. Moreover, thoracic PVAT from sedentary high-fat diet-fed rats shows a potent anti-contractile effect toward serotonin that was associated with an increased expression of inducible NOS (iNOS) [39]. However, a loss of function analysis of iNOS must be carried out to explicitly clarify its role because iNOS overexpression in thoracic PVAT of obese mice is also associated with endothelial dysfunction [65].

**Figure 2:**
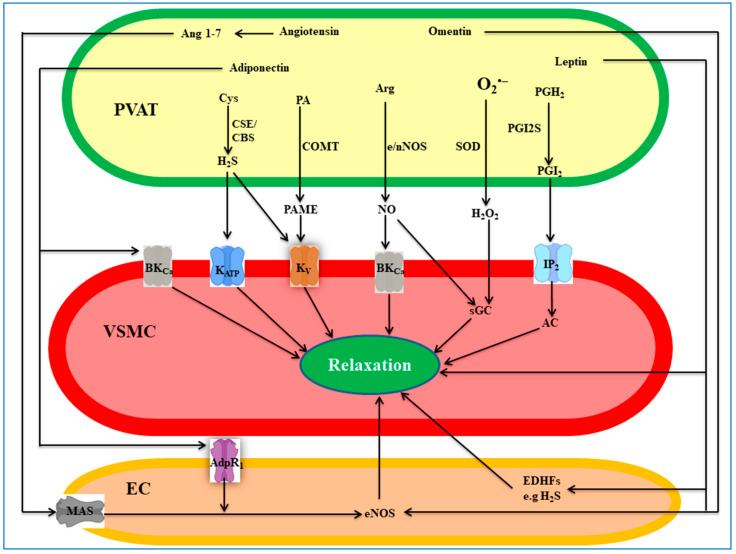
An overview of PVAT-derived anti-contractile factors and the mechanisms underpinning their effects. PVAT synthesizes several factors, including gases (NO and H_2_S), small molecules (H_2_O_2_, PGI_2_, and PAME) and proteins or peptides (adiponectin, angiotensin 1-7, leptin, and omentin). These factors act directly on VSMCs or indirectly on ECs through various mechanisms, such as activating K^+^ channels, soluble guanylyl cyclase, and prostacyclin I_2_ receptors or by activating eNOS, ultimately reducing VSMC contraction. EC, endothelial cell; PVAT, perivascular adipose tissue; VSMC, vascular smooth muscle cell; AdpR1, adiponectin receptor 1; BK_Ca_1.1, Ca^2+^-activated K^+^ channel; IP2, prostacyclin I_2_ receptor; K_ATP,_ ATP-sensitive K^+^ channel; K_V_, voltage-gated K^+^ channel; AC, adenylyl cyclase; CBS, cystathionine β-synthase; COMT, catechol-O-methyltransferase; CSE, cystathionine γ-lyase; e/nNOS; endothelial or neuronal nitric oxide synthase; PGI2S, PGI_2_ synthase; sGC, soluble guanylyl cyclase; SOD, superoxide dismutase; Ang 1-7, angiotensin 1-7; Arg, arginine; Cys, cysteine; EDHF, endothelium-derived hyperpolarizing factor; NO, nitric oxide; PA, palmitic acid; PAME, palmitic acid methyl ester; PGH_2_, prostaglandin H_2_; PGI_2_, prostacyclin I_2_.

Mechanistically, PVAT-derived NO diffuses to VSMCs, where it elicits vasodilation by directly activating K_Ca_1.1 channels and/or indirectly activating the sGC-cGMP pathway, thus causing membrane hyperpolarization (Figure 2) [66,67]. Additionally, adipocyte-derived NO stimulates the secretion of the anti-contractile adiponectin [68]. PVAT eNOS is also susceptible to uncoupling and hence reduced NO production under oxidative stress conditions, e.g., when NADPH oxidase activity increases and/or ROS scavenging enzymes decrease, as occurs in obesity [57,68]. In conclusion, NO is a bona fide PDRF that plays a key role in the regulation of vascular tone, including small vessels suggesting a critical role of PVAT-derived NO in the regulation of blood vessels. PVAT-based therapeutics strategies targeting NOS activity and reducing its uncoupling should be considered in future studies.

### 4.2. Hydrogen Sulfide

H_2_S is a gasotransmitter synthesized endogenously from L-cysteine by the action of two pyridoxal 5-phosphate-dependent enzymes, namely cystathionine β-synthase (CBS) and cystathionine γ-synthase (CSE) or by 3-mercaptopyruvate sulphurtransferase (MPST) [69]. In the cardiovascular system, H_2_S, produced in the endothelium and VSMCs mainly by CSE, causes vasodilation either by activating endothelial intermediate- and small-conductance Ca^2+^-activated K^+^ channels, which hyperpolarize the underlying VSMC membrane, or by directly stimulating VSMC ATP-sensitive K^+^ channels and inhibiting Ca_V_1.2 channels [70,71,72]. PVAT expresses CSE and CBS capable of synthesizing H_2_S [73], which exerts a potent anti-contractile effect in rat aorta [74,75], *gracilis* [76], and mesenteric arteries [74,77]. This effect is mediated by ATP-sensitive K^+^ [75] or XE991-sensitive KCNQ channels [48,74,78] and significantly antagonized by the CSE inhibitors DL-propargylglycine and β-cyano-L-alanine [69,70,71,72,73] (Figure 2). PVAT surrounding porcine coronary arteries antagonizes the hypoxia-induced contraction and potentiates relaxation induced by hypoxia through mechanisms involving the CBS-H_2_S pathway [79]. H_2_S mediates the anti-contractile effect of nucleoside 5′-monophosphorothioates (AMPS and GMPS) in PVAT-intact but not in PVAT-denuded aortic rings [80]. The loss of PVAT anti-contractile effect in mesenteric arteries of rats fed a high-fat diet is restored by physical exercise, which increases H_2_S production [81]. Moreover, the lipophilic atorvastatin augments PVAT anti-contractile effect in rat aorta by decreasing the coenzyme Q9 level and hence mitochondrial oxidation of H_2_S [82,83]. Taken together, H_2_S is a validated anti-contractile factor produced in PVAT of both small and large vessels that crucially contributes to its anti-contractile effect. Increasing H_2_S production or decreasing its metabolism can be exploited as a new therapeutic strategy for the treatment of several cardiovascular comorbidities.

### 4.3. Hydrogen Peroxide

H_2_O_2_, a small, non-free radical member of reactive oxygen species (ROS), is recognized as a pivotal mediator of oxidative signalling [84]. In vasculatures, H_2_O_2_ is produced both in the endothelium and VSMCs by superoxide dismutases and plays a critical role in cardiovascular physiology and pathology [85,86]. The effect of H_2_O_2_ on vascular tone is controversial. On the one hand, H_2_O_2_ induces vasodilation in different vascular beds via the activation of K_ATP_ and 4-aminopyridine-sensitive K_V_ channels [87,88], increasing PKG Iα dimerization and activation [89] and prostacyclin release [90,91]. On the other hand, it can also trigger vasoconstriction by inducing thromboxane A2 (TXA_2_) generation in VSMCs [92,93] and activating PKC and the IP3 pathway [94]. PVAT produces H_2_O_2_ that has been associated with an anti-contractile effect in several experimental models. In this context, Gao et al. [9] showed that PVAT anti-contractile effect against phenylephrine-mediated contraction involves both an endothelium-dependent and -independent mechanism, the latter being mediated by the H_2_O_2_ activation of the sGC-cGMP pathway [9]. In addition, mitochondria-derived H_2_O_2_ contributes to PVAT anti-contractile effect in rat thoracic aorta upon noradrenaline stimulation [95]. PVAT surrounding rat mesenteric arteries shows a potent anti-contractile effect towards noradrenaline involving not only neurotransmitter uptake and metabolism but also H_2_O_2_ release [96]. Additionally, nNOS-derived H_2_O_2_ is critical for PVAT anti-contractile effect in Balb/mice thoracic aorta [63]. Of interest, a high carbohydrate diet induces a potent anti-contractile effect of PVAT towards phenylephrine, in which H_2_O_2_ appears to play a key role [97]. Similarly, PVAT-derived H_2_O_2_ protects against vascular endothelial dysfunction caused by a single, high dose of ethanol [98]. The use of the H_2_O_2_ scavenger catalase provided indirect evidence of the involvement of PVAT-derived H_2_O_2_ in the potentiation of propofol-induced relaxation of rat thoracic aorta rings [99] and of proteinase-activated receptor-2 (PAR2)-active peptides SLIGRL-NH2 and 2-furoyl-LIGRLO-NH2-induced relaxation of both lean and obese mice aortic rings [100]. However, in diabetic rats [101] or obese mice [16], PVAT-derived H_2_O_2_ mediates a contractile effect, thus increasing vascular tone. All the aforementioned studies suggest that PVAT-derived H_2_O_2_, produced by either mitochondria or cytoplasmic pathways, diffuses down to VSMCs to mediate endothelium-independent PVAT anti-contractile effects, the final effectors being sGC activation, and K^+^ channel stimulation (Figure 2). However, under certain pathological conditions, H_2_O_2_ may contribute to PVAT contractile effect. Further studies are needed to clarify the controversial role of H_2_O_2_ in PVAT regulation of vascular tone and to quantify its contribution to PVAT anti-contractile effect in small vs. larger arteries before any H_2_O_2_-based drugs can be appreciated.

### 4.4. Prostanoids

Prostanoids are a class of arachidonic acid-derived bioactive lipids that include prostaglandins (PGE2, PGD2, PGF2α, and TXA2) and prostacyclin [102]. In the vasculature, both PGE2 and prostacyclin PGI2, produced mainly by the endothelium, induce potent vasodilation of the underlying VSMCs by activating the EP/IP receptors-adenylyl cyclase-cAMP pathway (Figure 2) [103,104]. The involvement of prostacyclin in PVAT anti-contractile effect is still a matter of debate. Prostacyclin synthesis inhibition by the nonspecific cyclooxygenase (COX) inhibitor indomethacin and the selective blockade of the PGI2 receptor antagonist Ro1138452 reduce PVAT anti-contractile effect toward phenylephrine-induced contraction in thoracic aorta of both male Wistar–Kyoto [105] and male Wistar Hannover rats subjected to sepsis [106]. In the latter model, a high level of both superoxide anion and 6-keto-PGF1α (a stable product of prostacyclin) was detected in PVAT [106]. These effects were also reproduced in the human saphenous vein, where indomethacin blocked the increase in PVAT PGE2 levels and decreased PVAT anti-contractile effect toward noradrenaline [107]. PVAT-derived prostacyclin prevents endothelial dysfunction in high-fat diet-fed C57BL/6J mice [35]. In mesenteric arteries from spontaneously hypertensive, obese and Wistar-Kyoto rats, PVAT shows a potent COX-2 activity and releases PGE2, PGI2, and TXA2 (the latter is a pro-contractile factor) [108]. Moreover, endothelium-derived prostacyclin partially contributes to PVAT anti-contractile effect toward noradrenaline in mice mesenteric arteries [47]. However, other studies failed to detect a role for prostanoids in PVAT-mediated anti-contractile effect both in experimental models [8] and human arteries [41,108]. Taken together, these observations suggest that PVAT-derived prostanoids contribute to its vasoregulatory activity. However, much effort is necessary to clarify the discrepancies arising from the use of different animal models, vascular beds, health status, and vasoconstricting agents.

### 4.5. Palmitic Acid Methyl Ester

Only a few studies have investigated PAME, a potent vasodilator released from the sympathetic ganglion [109], which is synthesized by catechol-O-methyltransferase (COMT) from palmitic acid. Both 3T3-L1 adipocytes and rat aortic PVAT express membrane-bound and soluble-COMT. Noticeably, the levels of these enzymes are significantly reduced in SHR, suggesting a role for PAME in hypertension [110]. PAME was first identified as PDRF released spontaneously and Ca^2+^-dependently in aortic PVAT of Wistar–Kyoto rats, where it induces vasodilation by the opening of K_V_ channels [111]. Recently, Wang and coworkers [112] showed that PVAT surrounding rat aorta exhibits a potent anti-contractile effect against serotonin-induced contraction, which was abolished by the K_V_7 channel blocker XE991. Further analysis showed that PAME is released from PVAT in response to serotonin stimulation, thus challenging the hypothesis of spontaneous release of PAME reported earlier [111]. These studies suggest that PAME is a PDRF, which contributes to PVAT anti-contractile effect via stimulation of K_V_ channels (Figure 2). However, due to the limited evidence, the role of PAME in PVAT regulation of vascular tone requires further studies, particularly in small blood vessels.

### 4.6. Angiotensin 1-7

Angiotensin 1-7 is a heptapeptide produced by the cleavage of angiotensin I or angiotensin II and counterbalances almost all physiological effects of angiotensin II [113]. PVAT contains all the actors involved in angiotensin 1-7 synthesis [angiotensin I, angiotensin II, and angiotensin-converting enzyme 1/2 (ACE1, ACE2)] [114,115]. The role of angiotensin 1-7 as PDRF has been demonstrated in rat aorta [116,117], rat inferior *vena cava* [118], and mice aorta [63,119]. It is responsible for the endothelium-dependent component of the anti-contractile effect of PVAT [116,117]. In fact, donor solution from PVAT intact vessels induces relaxation only in recipient tissues with an intact endothelium, and this effect is abolished by the eNOS inhibitor L-NAME, NO scavengers, the Mas receptor blocker A779, and the ACE2 inhibitor DX600 [116,117,119]. Angiotensin 1-7 exerts its effect by activating the G protein-coupled receptor Mas [120], which is expressed in endothelial cells, VSMCs, and PVAT [20,119,121]. The vasodilation induced by angiotensin 1-7 is mediated either by endothelial cell Mas receptors that, in turn, activate eNOS via the PI3K/Akt pathway to produce NO, which hyperpolarizes the underlying VSMCs through the activation of K_V_ channels [117,118,119] or by PVAT receptors leading to activation of eNOS and nNOS. The latter produces NO and H_2_O_2_, both of which are known as PDRF (Figure 2) [63]. Whether PVAT-derived angiotensin 1-7 acts directly on VSMCs is still unknown. In summary, there is enough evidence proving the role played by angiotensin 1-7 in PVAT anti-contractile effect. Therefore, a fruitful therapeutic approach should develop drugs capable of increasing angiotensin 1-7 production and stability or stimulating tissue-specific Mas receptors for the treatment of PVAT dysfunction-associated diseases.

### 4.7. Adiponectin

Adiponectin, secreted mainly by adipose tissues, is an adipokine that regulates several physiological functions, including metabolism and vascular tone. This is accomplished through the binding to and activation of adiponectin receptors AdipoR1 and AdipoR2 [122,123]. Initially, Fésüs et al. [124] showed that exogenous adiponectin reduces serotonin-induced contraction by activating K_V_ channels. However, the anti-contractile effect of PVAT in adiponectin gene-deficient mice is similar to that of wild-type animals, suggesting that it cannot be considered a PDRF candidate, though other compensatory mechanisms responsible for PVAT anti-contractile effect in adiponectin knockout mice should not be ruled out. In fact, this hypothesis has been challenged by several studies showing that this hormone is a local vasodilator and a PDRF [47,59,125]. For instance, Wither et al. [126] observed that PVAT anti-contractile effect is lost in eosinophil-deficient mice and is restored after eosinophil reconstitution. The anti-contractile effect was due to adiponectin and NO released from adipocytes upon activation of adipocyte β_3_-adrenoceptors by eosinophil-derived catecholamines. Indeed, under basal conditions, PVAT-intact mouse mesenteric arteries release, upon β_3_-adrenoceptors stimulation, a hyperpolarizing factor, probably adiponectin, capable of hyperpolarizing the underlying VSMCs via the opening of BK_Ca_ 1.1 channels and NO release from adipocytes [66]. Furthermore, in mice fed a high-fat diet, AMPK phosphorylation and adiponectin secretion are reduced, and the anti-contractile effect of aortic PVAT is lost, similarly to AMPK α_1_ knockout mice [127,128]. In addition, in obese mice, the anti-contractile effect of femoral artery PVAT is markedly reduced, as is the acetylcholine-induced relaxation. This effect was ascribed to a reduction in the level of p-eNOS^Ser1177^, Cu/Zn-SOD, and adiponectin levels as well as AdipoR1 expression along with increased leptin and ROS production, and was reversed by exercise, suggesting a crucial role of the adiponectin-AdipoR1 pathway in PVAT anti-contractile activity [129]. A similar dysfunction was reported in obese rat models [130,131]. In fact, adult male offspring of mice that experienced gestational intermittent hypoxia (GIH), for example, exhibit a loss of PVAT anti-contractile effect in the abdominal aorta that was rescued by exogenous application of adiponectin [132] or by the isoflavonoid calycosin through upregulation of the adiponectin/AMPK/eNOS pathway [133].

Different mechanisms underlie adiponectin-induced vasodilation: stimulation of eNOS activity and biosynthesis of its substrate tetrahydrobiopterin in endothelial cells [134]; stimulation of VSMCs directly by activating BK_Ca_ 1.1 or Kv channels or indirectly through NO release from adipocytes [47,59,68,125]; and inhibition of VSMC NADPH oxidase via PI3K/Akt-mediated block of Rac1 and down-regulation of p22phox gene expression (Figure 2) [135]. All the aforementioned studies suggest that adiponectin contributes to the anti-contractile effect of PVAT. As its function is reduced in obese models, adiponectin-adipoR1/2 pathways can be considered a potential target for the treatment of obesity-associated comorbidities.

### 4.8. Leptin

Leptin is a hormone produced mainly by adipose tissue to regulate appetite and energy expenditure. Under physiological conditions, it stimulates the sympathetic nervous system (indirectly causing vasoconstriction) and directly induces vasodilation: both processes are generally balanced with no net change in vascular tone [136]. Leptin-induced vasodilation may occur through either eNOS activation [137] or endothelium-derived hyperpolarizing factor (e.g., H_2_S) release [138]; an endothelium-independent VSMC hyperpolarization has also been observed (Figure 2) [139].

Though Löhn et al. [8] ruled out the involvement of leptin in PVAT-mediated anti-contractile effect, several studies have demonstrated that this hormone is expressed and released by PVAT of various vascular beds [140,141,142,143]. Gálvez-Prieto et al. [140] showed that PVAT and exogenous leptin markedly reduce angiotensin II contraction in normal but not in spontaneously hypertensive rats, an effect mediated by endothelial NO release. Interestingly, the enhancement of endothelium-dependent relaxation caused by PVAT-derived leptin involves the downregulation of 6-phosphofructo-2-kinase/fructose-2,6-bisphosphatase 3 (PFKFB3)- mediated endothelial glycolysis [142,143]. An increase in the NADPH oxidase subunit NOX1 expression and ROS generation seem to link glycolysis to the impairment of endothelial-dependent relaxation [144]. Furthermore, electrical field stimulation evokes a potent neurogenic relaxation in mesenteric arteries with intact PVAT compared to their PVAT-denuded counterpart. This relaxation was ascribed to leptin release from PVAT adipocytes and diminished upon low oxygen exposure [141]. Leptin levels increase during the early phase of diet-induced obesity and are linked to NO overproduction and protection against endothelial dysfunction [57]. Similarly, in Dahl salt-sensitive rats, aortic PVAT shows a potent anti-contractile effect toward phenylephrine, which is blunted by PVAT removal and eNOS inhibition, and mimicked by the addition of exogenous leptin [145].

In Ossabaw swine with metabolic syndrome, bradykinin-induced relaxation is reduced in coronary arteries with intact epicardial adipose tissue compared to denuded vessels and was linked to increased expression of leptin and its receptor along with protein kinase C β stimulation [146]. Several studies suggest that in obesity, PVAT loses its anti-contractile activity or even promotes endothelial dysfunction along with an increase in leptin levels. This phenomenon has been observed in aortic and femoral artery PVAT of obese mice and was associated with an increased expression of leptin, tumour necrosis factor α, iNOS, and monocyte chemoattractant protein [65,147] or reduced adiponectin secretion [129]. Similar findings have been reported in PVAT of small subcutaneous arteries of obese human subjects that were restored by bariatric surgery [148] and in small mesenteric arteries of obese rats that were reversed by weight loss [62]. Leptin can contribute to vascular dysfunction also by promoting VSMC phenotype switch [149,150] and neointima formation [143]. In fact, adipocytes release a high amount of leptin upon treatment with the hepatic fibroblast growth factor 21 (FGF21), suggesting the existence of a liver-PVAT-blood vessel axis [151]. These heterogeneous studies suggest that PVAT-derived leptin, under normal physiological conditions or during the early phase of obesity development, acts as a PDRF. However, in the context of obesity, it causes a pro-contractile effect through mechanisms that remain poorly studied. Further investigation is required to clarify the factors that determine the anti-contractile or pro-contractile activity of leptin before any therapeutic strategy is considered for the treatment of cardiovascular diseases.

### 4.9. Omentin

Omentin, a 34-kD adipokine produced mainly by visceral adipose tissues, exists in two isoforms, omentin-1 and omentin-2, sharing an amino acid identity of 83%: the former is predominant in human plasma [152,153]. Omentin-1 is expressed in different tissues, including epicardial fat, colon, thymus, small intestine, and ovary, and is identified by different names: intelectin-1, intestinal lactoferrin receptor, endothelial lectin HL-1 or galacto-furanose-binding lectin [152,154]. Omentin, which exerts anti-inflammatory, antioxidant, anti-atherogenic and cardioprotective functions [155,156], is expressed in PVAT. Furthermore, human perivascular pre-adipocytes release omentin upon stimulation with FGF21 [151]. Human epicardial adipose tissue surrounding large coronary arteries expresses omentin, and its level is lower in patients with coronary artery disease than in their healthy counterparts [157]. Similarly, PVAT of the human internal mammary artery expresses omentin, which exerts an anti-atherosclerotic function [158]. Direct evidence demonstrating the involvement of omentin in PVAT anti-contractile effect is still lacking, as PVAT-specific omentin knockout animal models are not available yet. Nevertheless, human recombinant omentin relaxes noradrenaline-induced contraction of both rat aorta and mesenteric arteries in an endothelium-dependent manner via eNOS^Ser1177^ phosphorylation and independently of Akt or tyrosine kinase activation (Figure 2) [159]. In summary, the contribution of omentin to PVAT anti-contractile effect awaits more experimental evidence, particularly that involving animal models with omentin gene-specific deletion in PVAT.

### 4.10. Other Factors/Mechanisms

Besides those discussed above, there are several endogenous factors endowed with vasodilatory effects, including ghrelin [160], apelin [161], adrenomedullin [162,163], irisin [164,165], vaspin [166], sulphur dioxide [167,168], and carbon monoxide [169,170], among others. Whether they are produced in PVAT and contribute to its anti-contractile effect has not been investigated yet. Other mechanisms have been claimed as potential contributors to the anti-contractile effect of PVAT: for example, the re-uptake of catecholamines into PVAT may underpin its anti-contractile effect by decreasing the amount of noradrenaline reaching VSMCs and increasing the release of vasodilators (NO and adiponectin) by stimulating β_3_-adrenoreceptors in PVAT [96,171,172]. Extracellular vesicles (small membraneous particles formed by cells and released to transfer biological messages to neighbouring cells to influence their physiology and function) containing microRNAs, such as miR-221-3p released from PVAT of obese mice, evoke an inflammatory response in VSMCs promoting their proliferation and migration [173]. Whether these or other yet unidentified mechanisms play a key role in PVAT regulation of VSMC tone has to be clarified with further investigation.

## 5. PVAT-Derived Pro-Contractile Factors

### 5.1. Superoxide Anion

Superoxide anion is a free radical oxygen species involved in several patho-physiological processes [174]. At the vascular level, it is produced in the endothelium, VSMCs, and *adventitia* by the action of NADPH oxidase, xanthine oxidase, and by the mitochondrial electron transport chain [175]. Gao et al. [176] showed for the first time that superoxide anion is responsible for PVAT potentiation of superior mesenteric artery response to electrical field stimulation. Several pieces of evidence supported this hypothesis: fluorescent labelling with dihydroethidium detected superoxide anion in PVAT-intact rings, isolated PVAT, and PVAT-derived adipocytes; this potentiation was mimicked by the exogenous superoxide donor pyrogallol and significantly reduced by SOD, NADPH oxidase inhibition, and indomethacin; and it was not observed in PVAT-denuded rings [176]. However, this result should be considered with caution because the study employed electrical field stimulation, a strong and not physiological stimulus. Nevertheless, in obese animal models, superoxide anion levels are high and contribute to the loss of PVAT anti-contractile effect, thus promoting vascular dysfunction [16,147,177,178,179,180]. Increased superoxide anion generation is also observed in PVAT of rats fed high-sugar diet for 12 weeks (a metabolic syndrome model); this effect was accompanied by an increase of O-linked β-N-acetylglucosamine (O-GlcNAc) modification of eNOS and by the loss of PVAT anti-contractile effect [181]. Of note, PVAT-intact aortic preparations stimulated by noradrenaline show an increased level of superoxide anion that was hypothesized to modulate vessel contraction directly or after dismutation to hydrogen peroxide [95]. Higher levels of superoxide anion are measured in PVAT of old male C57BL6/N mice as compared to young counterparts; this ROS was associated with enhanced arterial stiffness, though the contractile function was not directly assessed in this study [182].

**Figure 3:**
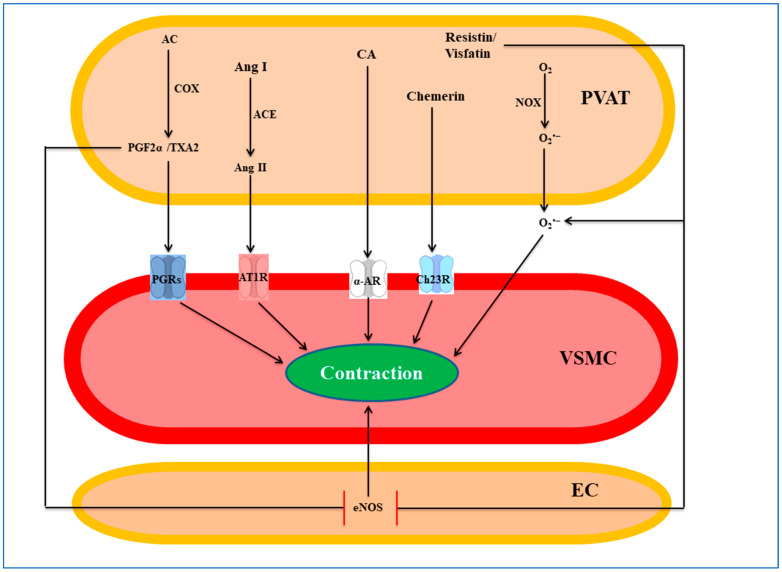
A synopsis of PVAT-derived contractile factors. PVAT synthesizes several factors and small molecules such as catecholamines and prostaglandins or peptides and proteins such as angiotensin II and chemerin. These factors act directly via different pathways on smooth muscle cells or indirectly by reducing the activity of eNOS, resulting in enhanced contractility of VSMCs. EC, endothelial cell; PVAT, perivascular adipose tissue; VSMC, vascular smooth muscle cell; αAR, α adrenergic receptor; AT1R, angiotensin II receptor type I; Ch23R, chemerin receptor 23; PGRs, prostaglandin receptors; ACE, angiotensin-converting enzyme; COX, cyclooxygenase; eNOS, endothelial nitric oxide synthase; NOX, NADPH oxidase; AC, arachidonic acid; Ang I, angiotensin I; Ang II, angiotensin II; CA, catecholamine; PGF2α, prostaglandin F2α; TXA, thromboxane A2.

The pro-contractile effect of superoxide anion is ascribed to the activation of the Rho-kinase pathway at the smooth muscle level [183], modulation of the arachidonic acid pathway [184], and/or NO inactivation to form peroxynitrite, which uncouples eNOS at the endothelial level [185]. Of note, PVAT eNOS uncoupling by superoxide anion has been reported in several studies [58,60,186,187]. Tyrosine kinase and the MAPK/ERK pathway are also involved as the inhibitors of both pathways (i.e., tyrphostin A25 and U0126, respectively) suppress the pro-contractile effect observed in PVAT intact preparations and attenuate the potentiation of the response operated by pyrogallol (Figure 3) [176]. Taken together, these studies suggest that NADPH oxidase and/or mitochondrial electron transport chain, under certain pathophysiological conditions, generate superoxide anion in PVAT that plays a pro-contractile function, thus potentiating the vascular response to various stimuli and reducing NO bioavailability.

### 5.2. Angiotensin II

Angiotensin II is a component of the renin–angiotensin system (RAS) capable of eliciting potent vasoconstriction, thus increasing blood pressure [188]. Shortly after the recognition of PVAT pro-contractile effect [176], PVAT was found to express complete RAS elements, including angiotensinogen, angiotensin II, angiotensin 1-7, ACE1, ACE2, renin, AT1R, and AT2R. PVAT RAS components vary across the vascular tree: angiotensin II level, for example, is higher in mesenteric than in periaortic PVAT [115,189]. In mesenteric arteries, PVAT-derived angiotensin II acts as a pro-contractile factor capable of potentiating the response to electrical field stimulation. In fact, both inhibition of angiotensin II synthesis by the ACE inhibitor enalaprilat and blockade of AT1R by candesartan blunted this effect [190]. The mechanism hypothesized involves PVAT-derived angiotensin II binding to adipocytes and vascular wall AT1R, stimulation of superoxide anion production by NADPH oxidase, and finally, vessel contraction through the activation of the tyrosine kinase-MAPK/ERK pathway (Figure 3) [191].

In a spontaneously hypertensive mouse model lacking perilipin-1 (Plin-1), a protein coating lipid droplets in adipocytes and regulating triglyceride storage and hydrolysis, high levels of angiotensin II, AT1R expression, and macrophage infiltration were detected in PVAT of both aorta and mesenteric arteries along with loss of anti-contractile effect and increased vasoconstriction [192]. More recently, adipose-tissue specific knockout of Bone Morphogenetic Protein 4 (BMP4) in apolipoprotein E (ApoE) knockout mice gave rise to high levels of angiotensinogen, angiotensin II, and ROS, resulting in hypertension development [193]. Furthermore, thiopental-induced relaxation of rat thoracic aorta is reduced by PVAT in an angiotensin II-dependent manner [194]. The use of AT1R antagonists supported the involvement of angiotensin II in several experimental models of diseases. For example, in high-fructose diet-fed rats, the AT1R antagonist losartan partially restored the endothelium-dependent PVAT anti-contractile effect, suggesting the involvement of angiotensin II in endothelial dysfunction [195]. In rat mesenteric arteries, the AT1R antagonist telmisartan and the ACE2 inhibitor captopril restore the anti-contractile effect of PVAT abolished by in vitro hypoxia, suggesting a role for angiotensin II in this phenomenon [196]. Furthermore, the blockade of both AT1R and AT2R restores the loss of PVAT anti-contractile effect in a rat model of heart failure caused by the overactivation of ACE1/angiotensin II/AT1R and AT2R pathway in PVAT [197]. Finally, angiotensin II has been linked to different pro-inflammatory phenotypes of PVAT that could be counteracted by PVAT browning [198]. Conversely, the contribution of angiotensin II to the loss of PVAT compensatory vasodilation during metabolic syndrome progression was ruled out as azilsartan, a potent AT1R antagonist, improves acetylcholine-induced vasodilation independently of the presence of PVAT [199]. Together, these studies demonstrate that PVAT, under certain pathophysiological conditions, synthesizes and secretes angiotensin II, which sequentially contributes to the loss of its anti-contractile activity by binding to AT1R expressed on adipocytes, VSMCs, and endothelium.

### 5.3. Prostaglandins

Prostaglandins, arachidonic acid-derived metabolites, are potent vasoconstrictors, capable of inducing also pro- and anti-inflammatory effects [200]. In the cardiovascular system, prostaglandins are produced not only by all blood vessel layers and cardiomyocytes [201] but also by PVAT. In experimental models of obesity, they play a different role. In obese mice, for example, prostaglandins significantly augment serotonin- and phenylephrine-induced contraction of the aorta while leaving unaltered that of the lean counterparts. This effect is blocked by COX inhibition or partially antagonized by inhibition of either COX-1 or COX-2 and is associated with elevated levels of TXA2 in periaortic PVAT [202]. PVAT-derived prostaglandins also cause endothelial dysfunction in mesenteric arteries of spontaneously hypertensive, obese rats but not in healthy controls. Again, this dysfunction can be reversed by the blockade of COX-2, TXA2 synthase, or PGI2 and TXA2 receptors [108]. Furthermore, in Cafeteria diet-induced obese rats, PVAT not only loses its anti-contractile but also exerts a pro-contractile effect associated with increased COX-1 activity as well as ROS, TXA2, and PGE2 levels [203]. Finally, minced PVAT constricts the thoracic aorta, carotid, and mesenteric arteries of C57BL/6J mice in an indomethacin-dependent manner [204].

Though PGE2 is a potent vasodilator, it can also elicit vasoconstriction under certain conditions, probably due to the existence of multiple receptors (EP1-4) coupled to different signalling pathways [205,206]. Ahmad et al. [207] showed that PVAT induces a pro-contractile effect in porcine coronary arteries, which was abolished by the COX inhibitors indomethacin and flurbiprofen. Interestingly, this study highlighted sex differences in PVAT function because the PGF2α receptor antagonist AL8810 attenuated PVAT-induced contraction only in males, while the TXA2 receptor antagonist GR32191B was effective only in female porcine coronary arteries. Furthermore, PGF2α levels were not statistically different between the two sexes: however, PGF2α elicited a stronger contraction in arteries from males compared to females, likely due to a higher prostaglandin F receptor expression. Conversely, TXB2 levels, a stable metabolite of TXA2, were significantly higher in females than males; however, neither the contraction evoked by the TXA2 agonist U46619 nor TP receptor expression were different between the two sexes [207]. Taken together, these studies suggest that PVAT synthesizes prostaglandins that, under certain pathological conditions, act as PDCFs, activating their receptors on VSMCs and counteracting its anti-contractile effect and/or mediating its vasoconstricting activity (Figure 3).

### 5.4. Catecholamines

Catecholamines are monoamine neurotransmitters endowed with several functions, such as regulation of metabolism and blood pressure [208]. Though these neurotransmitters are mainly produced by the sympathetic nervous system and adrenal medulla, endothelial cells are equipped with a complete system capable of synthesizing and secreting catecholamines in both in vitro and in vivo settings [209]. PVAT (e.g., aortic and mesenteric PVAT of male Wistar rats) also contains a reservoir of catecholamines, such as noradrenaline, dopamine, and serotonin [121,210], which are releasable by the indirect sympathomimetic tyramine, causing a greater contraction in arterial preparations with an intact PVAT as compared to those devoid of PVAT [211]. This tyramine-induced contraction is antagonized by the noradrenaline transporter inhibitor nisoxetine, the vesicular monoamine transporter inhibitor tetrabenazine, and the α-adrenoreceptor antagonist prazosin, but not by dopamine and serotonin transporters inhibition, or celiac ganglionectomy, suggesting that noradrenaline release does not occur in sympathetic neurons (Figure 3) [211].

The origin of catecholamines in PVAT is still a matter of debate. Indeed, PVAT may synthesize catecholamine de novo from their precursors, as adipocytes express all the necessary enzymes [212,213]. Alternatively, PVAT may take up catecholamine released from the sympathetic nervous system and store them. The following evidence support this hypothesis: accumulation in mesenteric PVAT is decreased by nisoxetine, by the serotonin transporter inhibitor citalopram, and by the organic cation transporter 3 inhibitor corticosterone, as well as by their combination [172]; PVAT surrounding mesenteric arteries of C57BL/6J mice exhibits an anti-contractile effect toward electrical field stimulation-induced contraction that is partially mediated by noradrenaline uptake into adipocytes [171]; PVAT adipocytes of thoracic aorta, resistance and superior mesenteric arteries take up noradrenaline via vesicular monoamine transporter 1/2, noradrenaline transporter, and corticosterone-sensitive organic cation 3 transporter [210]. Once into PVAT of rat mesenteric arteries, noradrenaline is metabolized by semicarbazide-sensitive amine oxidase and monoamine oxidase A; therefore, catecholamine metabolism can also affect PVAT anti-contractile effect [96]. Finally, other cells colonizing PVAT, such as macrophages and lymphocytes, may contribute to the pool, being capable of synthesizing catecholamines [214]. Eosinophils localized into PVAT, for example, synthesize and release catecholamines that, in turn, activate β_3_-adrenoreceptors on adipocytes, to release adiponectin and NO [126]. In conclusion, there is enough evidence supporting the existence of a catecholamines reservoir in PVAT, particularly noradrenaline, that potentially play a key role in anti-contractile and pro-contractile functions [211,215]. Whether PVAT catecholamines contribute to its pro-contractile effect under pathophysiological conditions deserves further investigation.

### 5.5. Chemerin

Chemerin is a multifunctional inflammatory adipokine, initially identified as a retinoic acid receptor response gene, which exerts its effect by binding to and activating the chemokine-like receptor 1, also known as ChemR23 [216], widely expressed in different organs and tissues, including vascular, endothelial, and smooth muscle cells [217]. PVAT localized to different vascular beds synthesizes and secretes chemerin [218,219,220]. Additionally, PVAT-derived pre-adipocytes and differentiated adipocytes secrete chemerin in conditioned media upon stimulation with FGF21 [151]. Chemerin and its active fragment chemerin-9 contract different vascular beds in a concentration-dependent manner [218,221,222,223]: this contraction is potentiated by endothelium denudation or eNOS inhibition [218]. Similarly, PVAT-derived chemerin potentiates phenylephrine-, prostaglandin F2α-, and electrical field stimulation-induced contraction of rat thoracic aorta, superior, and resistance mesenteric arteries: an effect blunted by the chemR23 antagonist CCX832 or in chemerin knockout rats [218,219,220].

Chemerin-induced contraction occurs through G_i_ activation leading to increased L-type Ca^2+^ channels opening and stimulation of Src kinase and Rho kinase activity [221,222]. PVAT-derived chemerin also contributes to obesity-induced endothelial dysfunction and hypertension [224,225]. These studies suggest that chemerin is a PDCF that might contribute to PVAT modulation of vascular tone (Figure 3). However, further studies are needed to define its contribution to vascular diseases and understand whether chemerin receptor antagonists might be valuable therapeutic weapons.

### 5.6. Resistin

Resistin, a small adipokine produced mainly by adipose tissues, is found in plasma as a trimer or hexamer. Once bound to receptors such as Toll-Like Receptor 4 (TLR4) or adenylyl cyclase-associated protein 1 (CAP1) [226,227], resistin triggers various intracellular signalling cascades leading to vascular inflammation, lipid accumulation, and oxidative stress [228]. Resistin was also found in the PVAT of different animal models under certain pathophysiological conditions [229,230,231,232]. PVAT-derived resistin increases the susceptibility to hypertension in males compared to female rats [233]. In this regard, PVAT-intact third-order mesenteric arteries from stroke-prone spontaneously hypertensive male rats do not relax to the K_ATP_ channel opener cromakalim as those from female counterparts: this effect was ascribed to the overexpression of resistin found only in male [233]. In support of this hypothesis, recombinant resistin impairs the response to cromakalim also in PVAT-intact vessels of female rats [233].

Resistin *per sé* neither causes vasodilation nor vasoconstriction. However, it attenuates insulin-induced vasodilation by inhibiting tyrosine/serine phosphorylation of insulin receptor substrate-1 and its sequential interaction with phosphatidylinositol 3-kinase, thus impairing Akt and eNOS phosphorylation [234,235]. Resistin also impaired the endothelium-dependent bradykinin- but not acetylcholine-induced relaxation of coronary artery rings, causing a reduction of NO and PGI2 synthesis and hence endothelial dysfunction [236]. Furthermore, resistin promotes the expression of endothelin-1, intercellular adhesion molecule-1 (ICAM-1), and vascular cell adhesion molecule-1 (VCAM-1) [237,238]. At the VSMC level, resistin significantly increases the phosphorylation of p42/4 mitogen-activated protein kinase (MAPK) and c-fos expression and downregulates the expression of cyclin-dependent kinases inhibitors (CDKIs) such as p53, p21, and p27 promoting cell proliferation [239,240]. In this regard, VSMCs cultured with either PVAT from obese mice or resistin alone show high levels of osteopontin, a key factor involved in VSMC proliferation, migration, and remodelling [229]. Though plasma resistin is mainly produced by monocytes and macrophages, at the cardiovascular level, PVAT resistin should not be underrated due to the proximity of PVAT to other vascular layers. Though the cellular origin of PVAT-derived resistin has not been investigated in depth, there is evidence that aortic PVAT of obese mice expresses high levels of visfatin and resistin, the latter being co-localized with macrophages [232]. In summary, though resistin cannot be considered as a standalone PDCF, it might contribute indirectly to PVAT pro-contractile activity or to the loss of its anti-contractile effect by inducing endothelial dysfunction and VSMC remodelling (Figure 3).

### 5.7. Visfatin

Visfatin is a multifunctional adipokine, produced mainly by visceral adipose tissues, also known as pre-B cell colony-enhancing factor (produced by lymphocytes), or extracellular nicotinamide phosphoribosyl transferase (eNampt), the limiting enzyme in nicotinamide adenine dinucleotide (NAD+) biosynthesis [241,242]. Visfatin, which can activate insulin receptors [243], and Toll-Like receptors [244], is associated with several diseases such as diabetes [245], obesity [246], and atherosclerosis [247], and its level increases during human pregnancy [248]. PVAT produces visfatin, and plasma visfatin level has been correlated with that of PVAT [232,249,250]. In addition, stimulation with FGF21 increases visfatin secretion from human perivascular adipocytes [151]. PVAT-derived visfatin can induce endothelial dysfunction and VSMC proliferation [232,249,250]. In this regard, Wang et al. [250] showed that PVAT-derived visfatin is ineffective on vascular tone induced by serotonin but rather acts as a growth-promoting factor for VSMCs. This notion, however, should be considered with caution. First, PVAT anti-contractile and pro-contractile effects are known to be influenced by the vasoconstricting agent used; in this study, serotonin was used [8,52]. Second, several studies have highlighted different effects of visfatin on vascular tone. For example, in rat aorta rings, visfatin antagonizes noradrenaline-induced contraction and induces endothelium-dependent relaxation by stimulating eNOS activity via phosphorylation at Ser^1177^ and de-phosphorylation of Thr^495^, independently of insulin receptor activation [251]. However, in rat endothelium-intact small resistance artery rings, visfatin does not alter noradrenaline response and markedly reduces the acetylcholine-mediated relaxation. This effect is reversed by either the NAMPT inhibitor FK866 or by superoxide dismutase, supporting a mechanism that involves ROS production and decreased NO bioavailability [249]. Similarly, visfatin attenuates acetylcholine- but not sodium nitroprusside-dependent relaxation in rat and human microvessels. This impairment is reversed by either the NADPH oxidase inhibitor apocynin or by the nicotinamide phosphoribosyl transferase inhibitor APO866 but not by an insulin receptor-blocking antibody, suggesting that visfatin-induced endothelial dysfunction occurs via a NAMPT/NADPH pathway [252]. In conclusion, PVAT-derived visfatin may contribute to its pro-contractile effect in small vessels: endothelial dysfunction and increased VSMC proliferation seem to play a key role in small resistance arteries (Figure 3). However, the evidence that, in large conduit arteries, visfatin may induce vasorelaxation via an endothelium-dependent mechanism indicates that further studies are necessary to clarify its interplay with PVAT function.

### 5.8. Other Factors/Mechanisms

Several other actors are believed to influence the contractile activity of PVAT. For example, PVAT attenuates adenosine-induced vasodilation by inhibiting K_Ca_ and K_V_ channels in lean Ossabaw swine and K_ATP_ channels in obese swine. Furthermore, exogenous calpastatin inhibits adenosine-induced vasodilation in lean but not in obese swine [49], likely as a consequence of the pro-contractile effect of coronary PVAT [50]. The role of calpastatin as PDCF and its mechanism has not been sufficiently addressed and deserves further attention.

Lipocalin-2, a pro-inflammatory adipokine upregulated during obesity and hypertension, has been associated with endothelial dysfunction [253]. A few studies show that PVAT expresses lipocalin-2, though the contribution to its pro-contractile effect has not been assessed yet [254,255]. Neuropeptide Y is a potent vasoconstricting agent released by the sympathetic nervous system [256], produced by the adipose tissue, where it regulates energy metabolism [257]. Whether PVAT can produce neuropeptide Y that contributes to its pro-contractile effect remains poorly studied. Tumour necrosis factor-alpha (TNFα), an inflammatory cytokine capable of potentiating VSMC contraction [258], is released by PVAT and contributes to the loss of its function [60,259]. Interleukins such as IL-6 and IL-1β, produced by many tissues, exert both pro- and anti-inflammatory effects and potentiate vessel contraction in diabetic and hypertensive rats [260,261]. PVAT secretes IL-6 and IL-1β, but their contribution to its contractile regulatory function is poorly understood [262,263]. Other factors detected in PVAT under pathophysiological conditions include platelet-derived growth factor-D [264], monocyte chemoattractant protein-1 [265], complement system components [266], and aldosterone [267]. However, whether these factors are involved in the PVAT pro-contractile effect or loss of anti-contractile function is not known yet and deserves further investigation.

## 6. Conclusions and Future Perspectives

PVAT is regarded as a metabolically active, endocrine organ capable of modulating vessel tone in many ways. Under physiological conditions, PVAT limits the contraction of blood vessels in response to various stimuli, thus protecting against hypertension and cardiovascular diseases. As this anti-contractile effect is mediated by a plethora of vasoactive molecules, a unique consensus on the role played by each of these mediators has not been reached yet, even in studies performed on the same blood vessel. This discrepancy may arise from the different conditions used to perform the experiments, including stimulating agents, age, sex, and health status of the animal model used. In addition, PVAT from different vascular beds of the same organisms behaves differently, likely because PVAT of different vessels exhibits different phenotypes and hence is definitively characterized by a unique secretory profile [268], though other factors (age, sex, stimuli, and health status) may also contribute. This problem might be circumvented by simultaneously assessing the PVAT anti-contractile effect of multiple vascular beds of the same animal model.

Under pathological conditions, particularly during the early stage of hypertension [77], obesity [97], and diabetes development [269], PVAT exerts a protective effect. However, once the disease is established, PVAT shifts from an anti-contractile to a pro-contractile phenotype by secreting numerous pro-contractile factors. In addition, in this context, a consensus on the role of the factors contributing to the loss of the anti-contractile effect is missing: standardization of the experimental conditions might help overcome this issue.

In this scenario of uncertainties, the precise role of PVAT-derived factors on vascular tone regulation should be unambiguously defined before PVAT-targeted therapeutics can be designed and developed. Therefore, future studies should systematically address the following issues. First, whether the list of PVAT vasoactive substances is complete or other factors/mechanisms are still to be discovered yet. Second, there is a need to investigate variability in PVAT anti- and pro-contractile factors depending on the animal models and vascular beds used, particularly in small vessels, which are known to play a key role in blood pressure regulation and hence hypertension pathogenesis. In this sense, it is also crucial to highlight the influence of stimuli, sex, age, and health status on the PVAT secretory profile. Third, taking the cellular heterogeneity of PVAT into consideration, the cellular origin of PVAT-derived factors needs to be clarified before any PVAT-target therapeutics can be designed. Fourth, further studies should also address the synthesis and release of PVAT anti- and pro-contractile factors and their regulation, as well as whether these factors are released in response to physiological or pathological stimuli or are constitutively produced. In addition, it is important to assess if these pathways work in vivo in the same way. During disease onset and progression, it is critical to determine the stage and mechanisms by which PVAT regulatory function shifts from an anti-contractile to a pro-contractile function and whether it can be reversed. Encouragingly, several studies have demonstrated the reversibility of PVAT function via weight loss [62], exercise [81,129], bariatric surgery [148], or small molecules [133]. Finally, the interplay between PVAT and factors released from the endothelium, VSMCs (myokines) or other adipose tissues (adipokines) under physiological as well as pathological conditions needs to be addressed.

Undoubtedly, the implementation of omic technologies is key to addressing these questions. Analysis of the transcriptome, proteome, metabolome, lipidome, and secretome of PVAT depots can identify the molecular hallmarks and the differences that may explain the discrepancies existing in the literature. Furthermore, the use of genome editing approaches such as knockout animal models for specific genes involved in biosynthetic pathways of PVAT vasoactive factors will definitively help to quantify their contribution to vascular tone regulation and identify any compensatory mechanism that may exist in certain gene knockout animal models.

## Data Availability

Not applicable.
